# The Maryland Dental Law

**Published:** 1892-01

**Authors:** 


### Editorial, Etc.

### THE MARYLAND DENTAL LAW.

In September, 1879, began the movement towards obtaining appropriate legislation for regulating the practice of dentistry in the State of Maryland. The sentiment then expressed was, "Require of the practitioner first a diploma, and then an examination; a diploma does not show that its holder is qualified to meet whatever demands may be made upon him in his profession."

In January, 1884, two bills were presented to the Legislature of Maryland. Both bills had in view the desire to awaken the public to the fact that they are daily preyed upon by persons who make pretensions to having what they do not possess, and were not framed for the pecuniary benefit of dentists as a class. One provided that "the board of examiners shall consist of five dental graduates appointed by the Maryland State Dental Association;" the other, for "a board of examiners to consist of five pracsicing dentists, appointed by the Governor from ten candidates, whose names shall be handed him by the Dental Legislative Society of Maryland." Neither bill was adopted in its entirety.

Nevertheless a dental law was passed and a board of dental examiners created "to consist of five reputable practicing dentists, appointed by the Governor, from the dentists residing in the State of Maryland." This act, approved March 31, 1884, made punishable only "willful" violations of the law. It called forth criticisms from the Pennsylvania State Dental Examining Board, the National Association of Dental Examiners, and several dental journals, because of a restrictive provision that "it shall be unlawful for any one to engage in the practice of dentistry unless he shall first have passed a satisfactory examination before the Board of Examiners or shall hold a diploma from a university or college chartered by or under the laws of Maryland." Exception was made in the act to persons who were engaged in the practice of dentistry in Maryland before its passage.

Acting upon these protests, the Dental Examiners of Maryland submitted amendments, approved by the dentists of the State, which placed the Maryland dental law in harmony with the dental laws of other States, and these the Legislature of 1886 passed without the change of a single word.

In 1888, the authorized code of public general laws superseded all the statute laws of the State of Maryland, and the dental law was again changed. The code, it has been construed, makes the following changes:

1. The law has no application to persons engaged in the practice of dentistry prior to March 31, 1884.

2. As to all other persons it is unlawful for them to practice without a certificate or diploma.

3. As the law now stands, it is uncertain as to cases occurring prior to the adoption of the Code whether they can get certificates by mere registration, or by examination before the board.

4. That one who is practicing without a certificate must be shown to have wilfully violated the law.

As the existing dental law is not expressed in precise, accurate terms about which there can be no dispute it has been proposed to have it amended. A bill was drafted and presented to the Legislature of 1890, but failed to reach a vote. The true reason for such laws is that the uniform experience of every civilized country has shown the wisdom and policy of securing the community against the impositions of ignorance and quackery. Such laws have been held to be constitutional, and the Supreme Court of the United States has affirmed the right of a State to regulate the practice of professions.

As the experience of eight years teaches that the dental law of Maryland has flaws and imperfections, and as the subject of dental legislation is of vital and practical importance to all dentists in the State, several desirable features are suggested to be embraced in the scope of the new act, which is intended to supersede the existing dental law.

1. A uniform standard of license should be established, so that if it is proposed to examine the dental graduates of any university or college chartered by the State, all should be included; although in eight years no diploma has been questioned, nor any college published as derelict by the Dental Examiners of Maryland.

A dental college diploma might exempt an applicant from examination, but it should not be made a prerequisite for examination. This is a rule in examinations for the bar, and it has worked satisfactorily.

2. Upon ordinary principles, in order to harmonize opposing interests in the organization of the new board, equal representation of each and every dental college or department should be recognized, as in Pennsylvania where the dental board is composed of three from the dental colleges and three from the profession at large. The duty of the board will be the protection of the public; the duty of each teacher in the board will be to guard the interests of his school.

3. Only reputable graduates in dentistry should be appointed by the Governor.

4. Examinations should be submitted in writing, and the questions and answers filed to be inspected by candidates and others interested.

5. The favorable vote of three members (if the board consists of five) should be sufficient to grant a permanent certificate. This power is a large one, but it is only fair to candidates whose examinations as to their knowledge and skill in dental surgery prove satisfactory to a majority of the board.

6. Provide that certificates shall be issued by the dental board only, and not by authority of the officers of the board or individual members of the board.

7. Exempt from registration non-resident dentists attending individual cases.

8. Provide for civil action against unlicensed practitioners to recover money paid by patient.

9. Forbid a minor to practice dentistry.

10. Forbid the assumption of the academic title "Doctor" unless conferred.

11. Forbid practicing under a diploma forged or bought.

12. Punish false personation of another practitioner.

